# Uncovering the biogeographic pattern of the widespread nematode-trapping fungi *Arthrobotrys oligospora*: watershed is the key

**DOI:** 10.3389/fmicb.2023.1152751

**Published:** 2023-04-20

**Authors:** Wei Deng, Fa Zhang, Yan-Peng Li, Xin Zhang, Davide Fornacca, Xiao-Yan Yang, Wen Xiao

**Affiliations:** ^1^Institute of Eastern-Himalaya Biodiversity Research, Dali University, Dali, Yunnan, China; ^2^Collaborative Innovation Center for Biodiversity and Conservation in the Three Parallel Rivers Region of China, Dali, Yunnan, China; ^3^The Provincial Innovation Team of Biodiversity Conservation and Utility of the Three Parallel Rivers Region, Dali University, Dali, Yunnan, China; ^4^Yunling Black-and-White Snub-Nosed Monkey Observation and Research Station of Yunnan Province, Dali, Yunnan, China

**Keywords:** research unit, fungi diversity, phylogeographic pattern, spatial distribution, river

## Abstract

Studies of biogeographic patterns of fungi have long been behind those of plants and animals. The presence of worldwide species, the lack of systematic sampling design and adequate sampling effort, and the lack of research units are responsible for this status. This study investigates the biogeographical patterns of *Arthrobotrys oligospora*, the most widespread globally distributed nematode-trapping fungi (NTF), by stratified collecting and analyzing 2,250 samples from 228 sites in Yunnan Province, China. The *A. oligospora* was isolated, and 149 strains were subjected to ITS, TUB, TEF and RPB2 gene sequencing and multi-gene association phylogeographic analysis. The results show that at population level *A. oligospora* is randomly distributed throughout Yunnan Province and has no biogeographical distribution pattern. At the genetic level, the phylogenetic tree of *A. oligospora* diverges into five major evolutionary clades, with a low degree of gene flow between the five clades. However, the correlation between the phylogenetic diversity of *A. oligospora* and geographical factors was low. There was no clear pattern in the phylogenetic clades distribution of *A. oligospora* either without dividing the study unit or when the grid was used as the study unit. When watersheds were used as the study unit, 67.4%, 63.3%, 65.9%, 83.3%, and 66.7% of clade 1–5 strains were distributed in the Jinsha river, Red river, Peal river, Lancang river, and Nujiang-Irawaddy river watersheds, respectively. The clades distribution of *A. oligospora* was highly consistent with the watersheds distribution. Training predictions of the clades distributions using randomly generated polygons were also less accurate than watersheds. These results suggest that watersheds are key to discovering the biogeographic distribution patterns of *A. oligospora*. The *A. oligospora* populations are blocked by mountains in the watershed, and gene flow barriers have occurred, which may have resulted in the formation of multiple cryptic species. Watersheds are also ideal for understanding such speciation processes, explaining factors affecting biodiversity distribution and coupling studies of plant and animal and microbial diversity.

## 1. Introduction

Biogeographic patterns and the mechanisms underlying them are essential components of biodiversity research. Understanding biogeographic patterns is crucial for predicting how global change affects biodiversity, designing effective conservation strategies, and achieving sustainable development (Gaston, 2000). Fungi are one of the most diverse taxa on Earth, widely distributed in various habitats around the globe, and play a critical role in ecosystems. However, their biogeography and the factors explaining their distribution pattern still need to be better understood (Martiny et al., 2006; Delavaux et al., 2019). The existence of clear non-ubiquitous biogeography for fungi has long been debated, with several reports showing weak patterns, often inconsistent with those of plants and animals or even bacteria (Martiny et al., 2006; O’Brien et al., 2016; Meyer et al., 2018). Addressing the current problems in the study of fungal diversity patterns is crucial to the study of biogeographic patterns of biodiversity as a whole. The following three problems are important reasons for the lagging behind in the study of biogeographic patterns of fungi.

Firstly, the need for more understanding of the genetic diversity patterns under fungal species is one of the reasons for their backwardness in research. Many worldwide species exist in fungal taxa, which may have undergone different historical evolutionary processes resulting in biogeographic pattern differentiation at the genetic level. Previous studies have identified this phenomenon in taxa such as *Aspergillus*, *Fusarium*, and *Saccharomyces* (Burt et al., 1996; O’Donnell et al., 2000; Matute et al., 2006). These species, which show differences in distribution at the genetic level, cannot be identified based on morphological studies alone. Due to the broader resolution of OTUs, it is also challenging to identify using only OTU-based amplicon sequencing techniques (Chase and Martiny, 2018). Recent developments in phylogeographic research have provided new ideas for this purpose. Focusing on the differentiation within widespread species and their biogeographical distribution at the phylogenetic level is expected to solve the above problems.

Another recognized issue affecting research in microbial biogeography is the need for systematic sampling design and insufficient sampling effort (Karimi et al., 2018). Most studies have been conducted based on amplicon sequencing technology in recent years. Researchers often believe that second-generation sequencing technology can detect almost all microorganisms in the environment and that sampling effort can be consequently reduced to save time and economic resources. However, numerous studies have demonstrated that inadequate sampling design and effort can introduce artifacts significantly affecting fungi and whole microbial diversity assessments (Hill et al., 1995; Castle et al., 2019; Hermans et al., 2019; Deng et al., 2020). Systematic deployment of sample sites and increased sampling efforts are undoubtedly necessary to address these problems.

Finally, the study of microbial biogeographic patterns needs a research unit. Mapping microbial diversity has been the goal of microbial ecologists (Martiny et al., 2006), and a suitable study unit is needed to map microbial diversity. An ideal study unit is an ecological unit with natural boundaries and geographic features essential for delineating the diversity range and mapping its distribution pattern. Current microbial diversity studies often do not specify a study unit or use an arbitrary grid as the analysis unit (Karimi et al., 2018; Ladau and Eloe-Fadrosh, 2019). This approach doesn’t properly consider geographical barriers such as mountains and rivers, creating confusion in the study by mixing the effects of multiple influencing factors.

Addressing the above three issues is the key to resolving the controversy of fungi biogeography. A watershed could be an appropriate natural unit. A watershed is the basic unit of the Earth’s terrestrial system, bounded by mountains and rivers, and is a relatively independent material and energy flow unit (Li et al., 2022). Watersheds bound the biogeographic provinces of many plants and animals (Brasil et al., 2017; García-Llamas et al., 2018). Watershed boundaries create barriers to fungi dispersal and help clarify confusion in current research.

*Arthrobotrys oligospora* Fres. is an ideal object for this study. *A. oligospora* is a species of Nematode-Trapping Fungi (NTF) from *Orbiliaceae*, Ascomycota (Niu and Zhang, 2011). This species can live both a saprophytic and predatory life. Its trophic hyphae specializes in forming three-dimensional adhesive networks to capture tiny organisms such as nematodes (Niu and Zhang, 2011). It is generally accepted that this species is widely distributed in various habitats worldwide and is a natural control factor for nematode populations in natural ecosystems (Niu and Zhang, 2011; Yang et al., 2011). The species also has many advantages as an object for microbial biogeographic pattern studies. *A. oligospora* is relatively easy to isolate and identify, its genome has been sequenced, and sequencing techniques are becoming more sophisticated (Yang et al., 2011; Jiang et al., 2018; Wang and Liu, 2023). Its widespread nature facilitates our studies at the level of phylogenetic diversity of its populations.

This research was conducted in Yunnan Province, China, which hosts the upper reaches of six major international rivers–the Lancang river, Irrawaddy river, Nujiang river, Red river, Pearl river, and Jinsha river–belonging to the Pacific and Indian Ocean watershed systems. The province covers an area of 394,100 km^2^ and spans an altitude of 76–6,740 m. The spatial heterogeneity in Yunnan is very high, particularly given that mountainous areas occupy 88.6% of the province. Previous studies found that many riverine ranges in southwestern China (including Yunnan) may have caused the unique differentiation of *A. oligospora*, confirming the feasibility of conducting this study in this region (Zhang et al., 2011). This study collected 2,250 samples from 228 sites in Yunnan, China, distributed in the six watersheds. The ITS (internal transcribed spacer region of the ribosomal RNA gene), TUB (beta-tubulin gene), TEF (translation elongation factor 1-alpha gene), and RPB2 (RNA polymerase II gene) fragments of the isolated *A. oligospora* strains were sequenced to map their genealogical and geographical distribution. We aimed to elucidate the phylogenetic diversity distribution pattern of the widespread microorganism *A. oligospor*a and to explore the suitability of watersheds as study units for microbial diversity.

## 2. Materials and methods

### 2.1. Research area

Yunnan province is located between 21^°^ 8’ 32”∼29° 15’ 8” N and 97^°^ 31’ 39”∼106^°^ 11’ 47” E, with a total area of 394,100 km^2^ and spans an altitude of 76–6740 m. The collision of the Indian Ocean and Eurasian plates largely shaped its geography. The subsequent formation of the Yunnan-Guizhou Plateau and the region’s folded landform explain its spatially heterogeneous landscape. Our study area is characterized by its sharp altitudinal gradient and the six large rivers–the Lancang river, Irrawaddy river, Nujiang river, Red river, Pearl river, and Jinsha river–cutting across the province. This complex topography helped shape the region’s relatively close ecological zones, where biodiversity is exceptionally high.

### 2.2. Sampling

**FIGURE 1:**
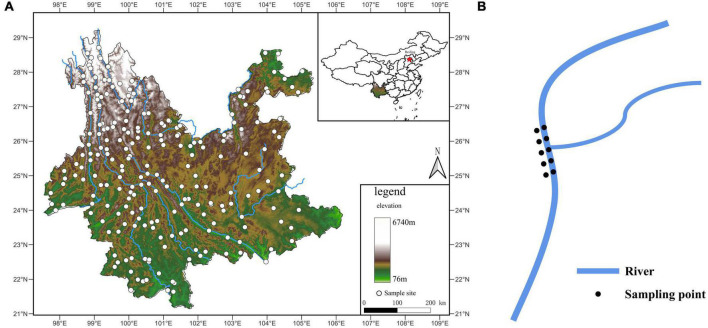
**(A)** Sample sites map. The white dots are sampling sites. The color change of the map represents the elevation change. The blue line is the rivers. **(B)** Sampling diagram. The blue line is the rivers.

We established 282 sampling sites within Yunnan’s hydrographic network. Sites included both terrestrial and aquatic habitats, and aquatic habitats included river stems and branches. We could not sample sites located in inaccessible terrain, including deep valleys and high mountains. Sites near cities with heavy human disturbance were also avoided. At the remaining 228 randomly distributed sites (Ripley’s K, *p* = 0.2435), we collected 2,250 specimens (Figure 1A). Sampling was performed between February 27 and June 8, 2014, starting from the southernmost to the northernmost locations to guarantee the sites’ phenological consistency and distinct aquatic-terrestrial border. Within each site, we sampled areas where two rivers joined. We considered the merging point as the origin, and a total of 10 samples of the origin, upstream and downstream, were sampled at a distance of 0 m, 10 m, and 20 m from the origin (samples included the aquatic and terrestrial habitats) (Figure 1B). Samples of mixed soil (or sediment) weighing 50 g each were taken using the five-point sampling technique at a depth of 0–10 cm. Samples were then stored in a disposable self-sealing bag at room temperature. We recorded habitat information, including each sampling site’s location, longitude, latitude, and elevation. Samples were sent to the laboratory within 1 week of collection, followed by *A. oligospora* phylogenetic identification process.

### 2.3. Isolation, purification, and morphological identification of *A. oligospora*

#### 2.3.1. Isolation and purification

Samples were plated on Petri plates with CMA media using the five-point spreading technique, and about 5,000 individuals of *Panagrellus redivivus* were added (Li et al., 2014). We plated three replicates per sample for a total of 6,750 Petri-dishes plated. The Petri dishes were incubated at room temperature (25–28^°^C). We examined the Petri dishes once after 3 weeks and again after 4–5 weeks using a stereomicroscope to isolate all the species (Deng et al., 2020). We used single spore isolation during each examination to collect and purify any germinated NTF on the CMA medium (Deng et al., 2020). A sterile toothpick was used during collection. Pure cultures were preserved until we could isolate and purify cultures from all specimens.

#### 2.3.2. Morphological identification

All preserved pure cultures were revived in CMA medium and placed on a temporary slide using the inserting and sticking method. Morphological characteristics of NTF, including conidia, conidiophores, and chlamydospores, were then photographed using a microscope camera (Olympus BX51, Japan). For each NTF strain, the type of trapping device was observed and confirmed using the observation chamber and nematode induction method. Strains were identified according to Hyde et al. (2014). A total of 662 strains were morphologically identified as *A. oligospora*.

### 2.4. DNA extraction, PCR amplification, and sequencing

One strain per sample site identified as *A. oligospora* was selected for subsequent analysis. It has been shown that the ITS (internal transcribed spacer region of the ribosomal RNA gene) sequences of some fungi can evolve rapidly and contain sufficient nucleotide sequence variation to be used for fine-scale studies under one species (Sha et al., 2008; Zhang et al., 2011). This study was based on ITS sequences, and given the multi-copy nature and combinability of ITS sequences, we additionally used a combination of TUB (β-tubulin gene), RPB2 (RNA polymerase II gene), and TEF (translation elongation factor 1-alpha gene) gene fragments for analysis (Zhang et al., 2011).

The pure culture was transferred to fresh PDA plates and incubated at 26^°^C for mycelium collection. The mycelium was collected using a sterile scalpel, and the genomic DNA was extracted using a rapid fungal genomic DNA isolation kit (Sangon Biotech Company, Limited, Shanghai, China). The Primer pairs ITS4-ITS5 (White et al., 1990), Bt1a-Bt1b (Glass and Donaldson, 1995), 526F–1567R (O’Donnell et al., 1998), and 6F–7R (Liu et al., 1999) were used to amplify the ITS, TUB, TEF, and RPB2 genes, respectively. The PCR amplification was performed in a 50 μL reaction system (2 μL DNA template, 3 μL of 25 mM MgCl_2_, 5 μL of 10 × PCR buffer, 1 μL of 10 μM dNTPs, 2 μL of each primer, and 1 unit of Taq Polymerase, and 34 μL ddH_2_O) under the following PCR conditions: 4 min pre-denaturation at 94^°^C, followed by 35 cycles of 45 s denaturation at 94^°^C, 1 min annealing at 52^°^C (ITS and TUB), 55^°^C (TEF), 54^°^C (RPB2), 1.5–2 min extension at 72^°^C, and a final 10 min extension at 72^°^C. The PCR products were purified with a DiaSpin PCR Product Purification Kit (Sangon Biotech Company, Limited, Shanghai, China). The purified PCR products of ITS TUB and RPB2 were sequenced in forward and reverse directions using PCR primers, and the primer pair 247F–609R (Yang et al., 2007) was used to sequence the TEF genes (BioSune Biotech Company, Limited, Shanghai, China).

### 2.5. Phylogenetic analysis

A total of 149 strains were involved in the subsequent analysis after the exclusion of low-quality sequenced strains. The sequences were checked and assembled using SeqMan Pro v.11 (DNAStar Lasergene, Madison, WI, USA). The assembled sequences were compared against the NCBI GenBank nucleotide database using BLASTn (accessed on 4 December 2022).^1^

The four sequences from each strain were summarized in a txt file and converted into a fasta file. MAFFT version 7 was used to generate the multi-sequence matrix (homologous sequences searching), and Bioedit was used to manually improve the alignment accuracy. We used the jModelTest v2.1.10 software to select the optimal calculating alternative model for the piecing sequence (ITS: GTR+I; TUB: SYM+I+G; TEF: GTR+I+G; RPB2: GTR+I+G). The phylogenetic tree was constructed following the Maximum Likelihood method (ML) in the way of partition calculation using IQ-Tree version 1.6.5 software. Post-processing and beautification of the phylogenetic tree with Interactive Tree Of Life (iTOL) v5 (Letunic and Bork, 2021).

### 2.6. Gene flow analysis of *A. oligospora* populations

The sequence fasta files were imported into PGDSpider 2.1.1.5 software after aggregation of the four sequences and the files were converted to MIGRATE format. The MIGRATE format file was read using migrate-5.0.4. software and the Bayesian method were selected for 10,000 iterations of gene flow analysis between 5 clades. Once the Bayesian Analysis: Posterior distribution table was obtained, gene flow was calculated according to the following equation.


Nm=θ×M÷x

where Nm is the gene flow, θ is the effective population size, M is the migration rate and x is the fixation factor. The four genes used in this study are nuclear genes, *x* = 4.

### 2.7. Biogeographical analysis of *A. oligospora*

The site occurrence frequency (OF _*site*_) of *A. oligospora* was calculated using the following formula.


$${\text{OF}}_{\text{site}}=\frac{\text{n}}{\text{N}}\times 100\%$$

where *n* is the number of sample sites where *A. oligospora* occurs and *N* is the total number of sample sites (i.e., 228).

The sample OF of each site was calculated using the following formula.


$${\text{OF}}_{\text{sample}}=\frac{\text{s}}{\text{S}}\times 100\%$$

where s is the number of samples where *A. oligospora* occurs in each site, and *N* is the total number of samples in the site (i.e., 10).

We mapped the distribution of *A. oligospora* populations using QGIS. Point pattern analysis of the distribution of the *A. oligospora* population in Yunnan Province was performed in R 4.1.2 using Ripleley’s K function, and the difference between it and random distribution was tested by chi-square.

The data of 19 climate factors from the worldclim website (accessed on October 20, 2022)^2^ were extracted using the raster package in R 4.1.2 according to the latitude and longitude of the sampling sites (Hijmans, 2022b). PCA analysis was performed to select four principal factors for subsequent analysis, bio4 (Temperature Seasonality), bio5 (Max Temperature of Warmest Month), bio15 (Precipitation Seasonality), and bio16 (Precipitation of Wettest Quarter) were selected.

Geographic distances between sample sites were calculated based on the sample site latitude and longitude using the geosphere package in R 4.12 (Hijmans, 2022a). Phylogenetic distances between sample sites were calculated based on the phylogenetic tree of *A. oligospora* using the picante package in R 4.12 (Kembel et al., 2010). Phylogenetic distances of *A. oligospora* were tested against geographic distances, elevation, and the above four climate factors using the Mantel test in the vegan package in R 4.12 (Dixon, 2003), and heat maps were plotted using the dplyr, linkET, ggplot2, and FD packages to present the results (Laliberte and Legendre, 2010; Laliberté et al., 2014; Wickham, 2017; Huang, 2021; Wickham et al., 2022). The R code used in this study can be found on GitHub.^3^

To test our hypothesis of the suitability of watersheds as spatial unit for microbial biogeography, we determined which phylogenetic tree branches of the *A. oligospora* strains isolated from each site was the most common in each watershed and calculated the error rate as the percentage of sites whose phylogenetic tree branch didn’t correspond to the majority of the respective watershed. We compared the accuracy of this approach with what would result from a simple distribution assessment using Euclidian distance. We generated Voronoi polygons around each site using a randomly selected portion of the sampling sites (training) and used the remaining sites for testing. Each created area represented the range of the phylogenetic tree branch identified in the respective site. This analysis was run 25 times using five iterations of five different training/testing dataset splits (55%, 65%, 75%, 85%, 95%) and the accuracy was assessed each time.

The difference between the accuracy rates when using the watershed as the unit and the Voronoi polygon was analyzed in R 4.1.2 using an independent samples *t*-test.

## 3. Results

### 3.1. Populations of *A. oligospora* are randomly distributed within Yunnan Province

**FIGURE 2:**
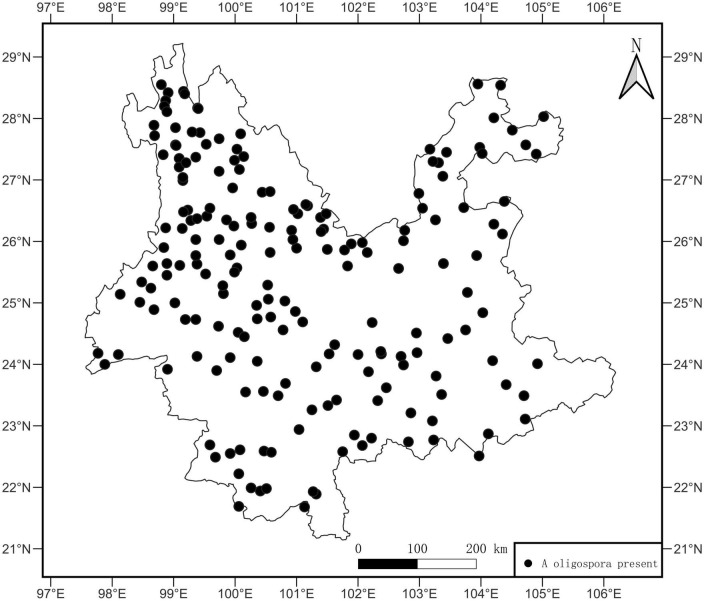
Distribution map of *A. oligospora*. The black dots in the map represent sample sites where *A. oligospora* was detected.

This study detected 662 strains of *A. oligospora* in 499 samples from 193 sites. The site OF was 84.65%. The sample OF for each site is shown in Supplementary Table 1, and the average OF for each site was 26.15%. *A. oligospora* was widely distributed in Yunnan Province, and the point pattern analysis showed a random distribution pattern (Figure 2, *p* = 0.48, X^2^ = 513).

### 3.2. Phylogenetic tree of *A. oligospora*, gene flow and the correlation of phylogenetic diversity and environmental factors

**FIGURE 3:**
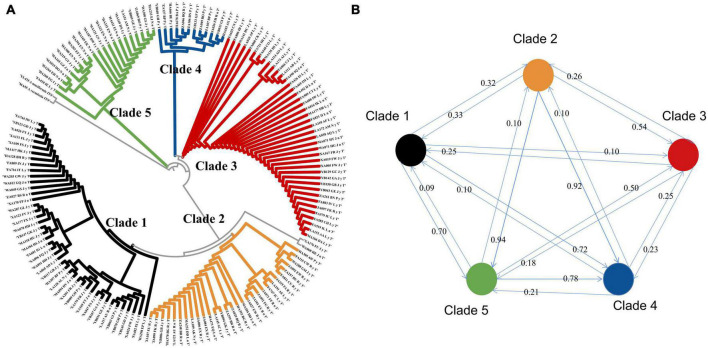
**(A)** Phylogenetic tree of *A. oligospora*. **(B)** Gene flow among the five clades of the *A. oligospora*.

The GenBank accession numbers of all the sequences of the 149 strains obtained in this study are shown in Supplementary Table 2. The phylogenetic tree demonstrates that the widespread species *A. oligospora* diverged into five large evolutionary clades (Figure 3A). The degree of gene flow between these five clades was low. None of the gene flow between the clades exceeded 1 (Figure 3B, mean 0.38, standard deviation 0.28). The lowest gene flow was from clade 1 to clade 5. The gene flow was 0.09. The highest gene flow was from clade2 to clade 5, the gene flow was 0.94.

**FIGURE 4:**
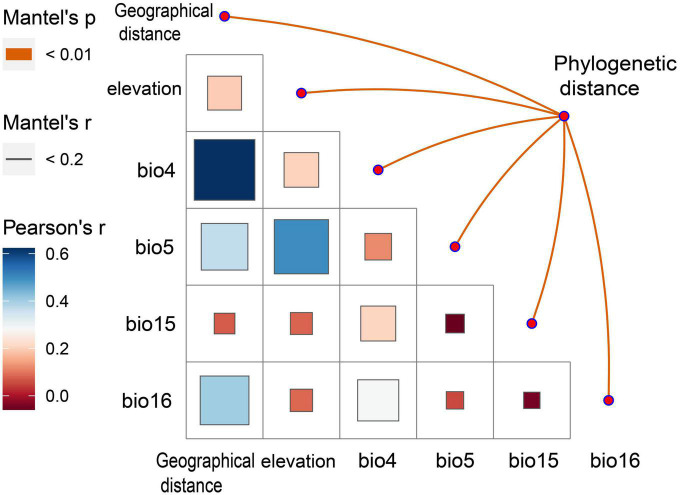
Mantel Test heat map between phylogenetic diversity of *A. oligospora* and geographic and climatic factors. bio4 is Temperature Seasonality, bio5 is Max Temperature of Warmest Month, bio15 is Precipitation Seasonality, bio16 is Precipitation of Wettest Quarter.

The results of the Mantel test showed a low correlation between the phylogenetic distance of *A. oligospora*, geographical distance, elevation, and the four climatic factors (Figure 4). All correlation coefficients were below 0.2. There is a strong autocorrelation between bio4, bio6, and geographical distance and between bio5 and elevation (Figure 4).

### 3.3. Watershed is the key to uncover the biogeographical pattern of *A. oligospora*

**FIGURE 5:**
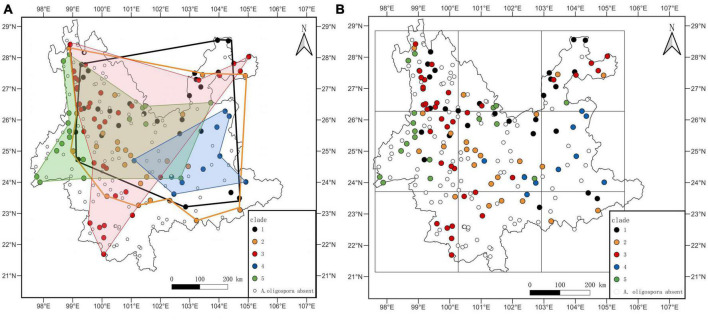
Map of the biogeographical distribution of *A. oligospora*; **(A)** is the map without the study unit, directly linking the individual Clade distribution boundaries; **(B)** is the map when the study unit is a grid.

When the distribution ranges of the individual clades are linked directly without dividing the study units, there is a heavy crossover between the distribution areas of the individual clades and no clear pattern. Clade 1 and clade 2 were mainly distributed in northern Yunnan (Figure 5A). Clade 3 is distributed throughout most of Yunnan except for southeastern Yunnan (Figure 5A). Clade 4 is distributed in eastern Yunnan, and clade 5 is distributed in northwestern Yunnan (Figure 5A). There was also no clear pattern in the distribution of Clades when the grid was used as the unit of study (Figure 5B). Strains from different Clades were mixed within each grid.

**FIGURE 6:**
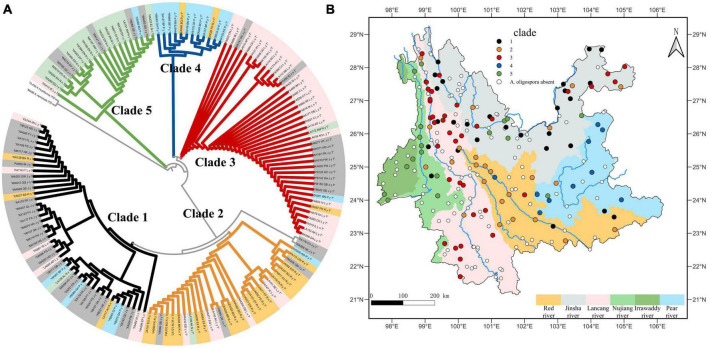
**(A)** Shows the phylogenetic tree of *A. oligospora*, with the color of the label of each node representing the watershed to which it belongs, consistent with the color of the watershed in **(B)**. **(B)** Shows the clades biogeographical distribution of *A. oligospora* when the watershed is used as the unit of study.

When using the watershed as the unit of study, the strains within each clade are largely confined to a single watershed, with the exception of the Irrawaddy, which does not have a corresponding clade. The strains in clades one through five were 67.4%, 63.3%, 65.9%, 83.3%, and 66.7% found in the Jinsha, Red river, Peal river, Lancang river, and Nujiang-Irrawaddy river watersheds, respectively (Figures 6A, B).

**FIGURE 7:**
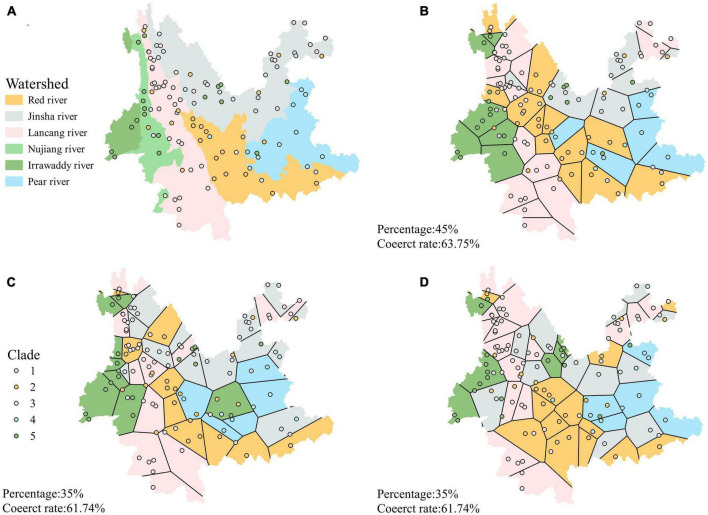
**(A)** Clade distribution map of *A. oligospora* with watershed as a unit. **(B–D)** Three maps with the highest accuracy when Voronoi polygons are the units.

The classification accuracy of the phylogenetic branches was 68% when the watershed was used as the unit (Figure 7A). In contrast, the average accuracy of the 25 predictions using Voronoi polygons was 50% (range: 36%–64%, mean 50%) (Table 1). The three maps with the highest accuracy when Voronoi polygons were used as units were 63.75% (Figure 7B), 61.74% (Figure 7C), and 61.74% (Figure 7D), respectively, but more than 35% of the sample points needed to be taken as the training set to obtain this accuracy. The highest accuracy was 64% for one map with Voronoi polygons, but the polygon distribution of this map was very similar to the watershed distribution (Figure 7). The difference of accuracies between the two approaches was significant (*p* < 0.0001, *t* = 10.79, df = 24, discrepancy = −0.18).

**TABLE 1:** Training prediction accuracy when Voronoi polygons are the units.

	Train	Test	Correct	Accuracy		Train	Test	Correct	Accuracy
V1	11	138	57	0.38	V14	38	111	74	0.50
V2	10	139	53	0.36	V15	38	111	72	0.48
V3	11	138	61	0.41	V16	55	94	89	0.60
V4	10	139	61	0.41	V17	53	96	92	0.62
V5	11	138	53	0.36	V18	53	96	90	0.60
V6	25	124	66	0.44	V19	54	95	83	0.56
V7	25	124	69	0.46	V20	55	94	82	0.55
V8	26	123	64	0.43	V21	70	79	85	0.57
V9	24	125	60	0.40	V22	65	84	70	0.47
V10	26	123	76	0.51	V23	58	91	95	0.64
V11	41	108	72	0.48	V24	62	87	92	0.62
V12	41	108	77	0.52	V25	62	87	76	0.51
V13	39	110	80	0.54	–	–	–	–	–
Mean	–	–	–	–	–	–	–	–	0.50
Median	–	–	–	–	–	–	–	–	0.50
Max	–	–	–	–	–	–	–	–	0.64
Min	–	–	–	–	–	–	–	–	0.36

## 4. Discussion

Nematode-trapping fungi (NTF) are natural limiting factors for nematode populations in ecosystems, and *A. oligospora*, the most studied and widely distributed species of NTF, has particular potential for development as a biocontrol agent (Niu and Zhang, 2011; Yang et al., 2011). Understanding its ecological characteristics and biogeographic pattern is significant for its conservation and exploitation. However, regarding ecological distribution, previous studies have only focused on NTF-preferred habitats and distribution in vertical soil layers (Dasgupta and Khan, 2015). Researchers have long believed that *A. oligospora* is widely distributed globally and has no biogeographical distribution pattern (Zhang et al., 2011). In the present study, *A. oligospora* was widely distributed in different habitats, and its populations did show a random distribution pattern. This result seems to support the hypothesis that *A. oligospora* has no biogeographical distribution pattern.

However, fungal diversity is extremely high. Recent studies have found that despite the slight morphological variation, many fungi have diverged at the infraspecific genetic level, and cryptic species have emerged within their species. These cryptic species often arise from geographical isolation and their neglect would obscure the biogeographical distribution patterns of the fungi. Ahren et al. (2004) found that the intraspecific genetic diversity of *A. oligospora* was greater than that of its close relative *Duddingtonia flagrans* based on 12 strains from different geographic regions, with potential geographic correlation (Ahren et al., 2004). Subsequently, Zhang et al. (2011) confirmed the high intraspecific variability of *A. oligospora* and identified three potential cryptic species within the species (Zhang et al., 2011). The results of the present study are consistent with these findings, with five large clades occurring within the *A. oligospora* species. The smaller gene flow in the population between these five clades also predicts the presence of more cryptic species within its species.

The study area of this study is located in the mountains of southwest China, an important biodiversity hotspot in the world. It seems inevitable that *A. oligospora* would be geographically isolated in this region and thus show a distribution pattern, as the Himalayan and Tibetan Plateau uplifts have created a complex landscape represented by the Three Parallel Rivers Region. However, the correlation between the phylogenetic diversity of *A. oligospora* and geographical distance, elevation and the four climatic factors is weak. When the study unit is not divided, or the grid is used as the study unit, the distribution areas of several clades overlap, making it difficult to see the distribution pattern.

The situation turned around when we used the watershed as the unit of analysis. Five clades of *A. oligospora* were distributed consistently in the six watersheds of Yunnan Province. The strains 67.4%, 63.3%, 65.9%, 83.3%, and 66.7% of clade 1–5 were distributed in the Jinsha river, Red river, Peal river, Lancang river, and Nujiang-Irawaddy river watersheds, respectively. The results of the training prediction analysis also showed that the accuracy of matching clades with geographic units was higher when using watershed units than randomly generated polygons. The three polygon maps with the highest accuracy also had polygons arranged in a way that was more consistent with the watershed distribution. These results suggest that watersheds are crucial to interpreting the biogeographic patterns of *A. oligospora* lineages. It also shows the possibility of watersheds as the best unit of analysis for studying microbial diversity and even the biogeographic pattern of biodiversity as a whole.

Watersheds have clear boundaries, and ecogeographical significance makes them natural division units for biogeographical analyzes. The dividing ridges between adjacent watersheds are critical dispersal barriers, and the relatively isolated materials and energy flow were shaped. Previous studies have considered watersheds as ecosystem boundaries and discussed the possibility of setting watersheds as the appropriate units of analysis in landscape ecology (Berkes et al., 1998). For example, García-Llamas et al. (2018) found that watersheds were better units when predicting diversity based on environmental heterogeneity (García-Llamas et al., 2018). The use of watersheds as a study unit for the distribution of fungi and biodiversity has several advantages.

Firstly, watersheds can help us understand the mechanisms of speciation. Allopatric speciation is an essential route to speciation, the central feature of which is the geographical separation of ancestral species populations into subpopulations and the emergence of gene flow barriers between subpopulations. Gene flow between the five clades in this study was low, while the distribution of the five clades corresponded well to the distribution of the watersheds. Thus, although *A. oligospora* is widely distributed throughout Yunnan Province, it is separated by mountains between watersheds, limiting gene flow between populations. These results support the speculation by Zhang et al. (2011) that *A. oligospora* is undergoing an allopatric species differentiation process and that the mountain between watersheds is an important barrier to gene flow in *A. oligospora* (Zhang et al., 2011). The watersheds, therefore, also play an essential role in understanding the mechanisms of allopatric speciation.

Secondly, Watersheds provide a natural study unit for understanding the factors determining biodiversity patterns. Understanding the distribution patterns of biodiversity and their underlying determinants is a fundamental topic in ecology and biogeography (Fine, 2015). Current research suggests that historical factors (e.g., geological history) and contemporary environmental conditions (e.g., climate) jointly shape biodiversity distribution patterns, yet their relative contributions are often difficult to distinguish (Kissling et al., 2012; Li et al., 2020). Watersheds provides a natural study unit for this purpose. Moving from large scales (secondary or tertiary watersheds) to more minor scales (sub-watersheds), ridge barriers’ influence on distribution gradually decreases while environmental influences increase. Therefore, the effects of historical events and contemporary environmental background are perfectly coupled by watershed units.

Thirdly, the watershed also offers the possibility of integrating the study of the three major taxa of organisms (animals, plants and microorganisms). A community in nature is a continuous unit that spans both temporal and spatial scales (Dasgupta and Khan, 2015). Any one community contains three major taxa of organisms, and communities of any size are highly complex. Current research often studies animal, plant, and microbial communities separately, which is detrimental to the overall community understanding (Dasgupta and Khan, 2015). However, the arbitrary division of study units also exists for plant and animal taxa. Biological provinces, the most commonly used units of study, do not correspond between plant and animal taxa either. Still less is there a unit of study that can be generalized between the three major taxa. The watershed provides a choice of units for biogeographic studies that apply to multiple taxa. In this way, the primary question of the influence of the coupling of the three major taxa in shaping biogeographic patterns as a basis for research.

In conclusion, watersheds as units will help to solve most of the problems of microbial diversity pattern studies in one package and are expected to be the best units in biodiversity studies.

## Data availability statement

The datasets presented in this study can be found in online repositories. The names of the repository/repositories and accession number(s) can be found below: https://www.ncbi.nlm.nih.gov/genbank/, OQ244107–OQ248207 (ITS), OQ266933–OQ267083 (TUB), OQ267084–OQ267234 (RPB2), and OQ267235–OQ267385 (TEF).

## Author contributions

WD, FZ, X-YY, and WX involved in the conception and design of the study. WD, FZ, Y-PL, XZ, and X-YY performed experimental work and collected the data. WD, FZ, DF, X-YY, and WX were data curation and interpretation. WD wrote the original draft of the manuscript. DF, WX, and X-YY contributed in terms of manuscript structuring and editing. All authors contributed to the article and approved the submitted version.

## Footnotes

1
https://blast.ncbi.nlm.nih.gov/Blast.cgi

2
https://worldclim.org/

3
https://github.com/dengweihx/Point-Pattern-Analysis-and-Mantel-test.git

## Supplementary material

Supplementary Table 1 The occurrence rate of *A. oligospora* of each site.

Supplementary Table 2 The GenBank accession numbers of the isolates included in this study.
